# Supplementary material for: An abdominal spacer that does not require surgical removal and allows drainage of abdominal fluids in patients undergoing carbon ion radiotherapy
Source: PLoS One. 2020 Jun 10;15(6):e0234471. doi: 10.1371/journal.pone.0234471 (PMC7286516; doi:10.1371/journal.pone.0234471)
Supplement: Attachment — Submitted filename: Response_to_reviewers.docx [file pone.0234471.s003.docx]

**RESPONSE TO REVIEWERS:**

Thank you for your constructive comments on our manuscript. The reviewers’ comments were very helpful. Our responses to the reviewers’ suggestions have been provided below. We wish to express our appreciation to the reviewers for their insightful comments, which have helped us significantly improve our manuscript.

Additional Editor Comments:
***①　Please note that only two animals were used for the purpose of this study. Can you provide more experimental data?***

**Response to the comment:**

It was a study to show the usefulness of the medical equipment that we newly developed. We did not evaluate the sample size for a significant difference. Furthermore, we restricted the laboratory animals to two for animal protection reasons.

***②　The Editors also noted that authors make strong claims over the results by using language such as "epoch making device".***

**Response to the comment:**

Thank you for your suggestion.

In accordance with the editor's comment, we have modified some text in the Discussion section as follows:

(Page 12, lines 197-200)

“The spacer may be a new medical device that can be converted from a spacer to a therapeutic drain during the therapeutic course of radiation depending on the patient’s status and the status of the abdominal infection.”

Furthermore, we have modified some text in the Conclusions section as follows:

(Page 15, lines 239-242)

“By using this new medical device, the indications for heavy ion beam therapy and IMRT may be extended for cancer patients at high-risk of abdominal infections, ruling out the use of existing balloons, Gore-Tex sheets, and absorbable spacer gels.”

***③　Authors must rewording the relevant sections to reflect the need of further studies prior to validation of the presented device.***
**Response to the comment:**

Thank you for your suggestion.

In accordance with the editor's comment, we have added the following sentence in the Discussion section:

(Page 14, lines 231-234)

“This study revealed the efficacy of our fan-shaped spacer for making distance between the target and surrounding organs and our spacer could be removed without difficulty. Nevertheless, long-term indwelling experiment, securing the durability and checking adverse events is necessary.”

Journal requirements:

*Please ensure that your manuscript meets PLOS ONE's style requirements, including those for file naming.*

**Response to the comment:**

We apologize for the oversight. We have changed the file naming according to PLOS ONE’s style.

***2. As part of your revision, please complete and submit a copy of the ARRIVE Guidelines checklist, a document that aims to improve experimental reporting and reproducibility of animal studies for purposes of post-publication data analysis and reproducibility:***

***Please include your completed checklist as a Supporting Information file.***

***Please also include in the Methods the type of anaesthesia administered to animals.***

**Response to the comment:**

We have attached the ARRIVE Guidelines checklist.

In accordance with the ARRIVE Guidelines checklist, we have revised the text in the Animal experiment section as follows:

(Page 6, lines 88-90)

“Two healthy pigs obtained from a three-way cross including the Landrace, Large White and Duroc came from the Fujii farm (Ishioka, Ibaraki, Japan). The pigs weighed 40 kg and each pig was used on a different day.”

Furthermore, we added the following in the Animal experiment section:

(Page 5, lines 91-92)

“We created a 3-cm laparotomy in the lower abdominal midline incision to insert the spacer during isoflurane anesthesia.”

Reviewers' comments:
Reviewer #1:
***Please check the manuscript for spelling mistakes (some words are missing).***

***Remove "infectious" from the title.
Please add a video of how you make/construct the spacer. This point is very important to reproduce the technique***

**Response to the comment:**

Thank you for your suggestion.

We have had a native English speaker proofread the manuscript again.

In accordance with the Reviewer's comment, we removed "infectious" from the title.

(Page 1, lines 1-2)

“An abdominal spacer that does not require surgical removal and allows drainage of abdominal fluids in patients undergoing carbon ion radiotherapy”

We have added the video of how the spacer was made.

In accordance with the Reviewer's comment, we added the sentences below under the “Structure of the novel fan-shaped spacer made of folded-film drain and two kinds of lines” subheading.

(Page 7, lines 104-132)

“Dots were plotted on a film tube to indicate the holes with distances of 40, 1.5, 2, 2, 2, 2, 1.5, 2, 2.3, 2.2, 2, 1.5, 2, 3, 3, 2, 1.5, 2, 3.5, 3.5, 2, 1.5, 2, 4, 4, 2, 1.5, 2, 4.5, 4.5, 2, 1.5, 2, 5, 5, 2, 1.5, 2, 5.5, 5.5, 2, 1.5, 2, 6, 6, 2, 3, 1, 3, 2, 13, 2, 3, 1, 3, and 2 cm in-between. Holes of 3.5 mm were made in the center of the film tube at the marked plots. A 3.5 mm hole was made 1 cm away from one end of the main drain. The film tube was inserted through the main drain from the side of the hole that was made. The first hole of the film tube was matched with the main drain’s hole, and the trigger line was inserted through the main drain. The trigger line was inserted into the third hole of the film tube, and every fifth hole of the film tube, creating loops on both the right and left sides of the trigger line (Fig 1b). The end of the trigger line was clipped to hold the loops.

A string was wrapped at the root of the trigger line, and both ends of the string were taken and threaded on a needle. One of the sides was picked and the needle was inserted through all the holes of the film tube on the trigger line side. One end of the string was taken and wrapped around the last hole once from the front and reinserted in the same direction as the needle passed through. The other end was taken and reinserted similarly from behind. Both ends of the string were tied. Another string was hung on the string at the root of the trigger line. Both ends of the string were taken, and the needle was threaded.

Similarly, it was inserted through all the holes on the film tube closest to the trigger line. As noted above, both ends of the string were inserted and the ends of the string were tied. Another string was hung on the root of the trigger line, both ends of the string were taken and a needle was threaded. The needle was inserted through the hole of the main drain. Then, the needle was inserted through the two lateral holes of the outermost loop of the film tube from the inside. From the outside, the needle was inserted through the remaining holes towards the root of the trigger line. The strings were reinserted and tied similarly. Another string was hung on the string where the outermost loop of the film tube was attached to the main drain. Both ends of the string were taken to thread the needle. The needle was inserted through the hole on the main drain, then the two lateral holes of the film tube from the inside, and then the remaining holes towards the root as with the other side. The unnecessary parts of the strings were cut. The trigger line was pulled so that the end would be inside the outer surface of the film tube (video).”

Reviewer #2:

***I read with interest the manuscript by Norio Kubo and collaborators about a new potential abdominal spacer to be used in patients undergoing radiotherapy. The authors show how this device acts both as a spacer, to perform local radiation of the tumor safely, as well as a drainage system to diagnose and treat eventual abdominal infections.
Additionally, the authors report that removal of this new fan-shaped spacer can be performed without surgery.
The authors should include some information about statistical methods they use and how they calculated their sample size. The study miss a control group, or alternatively, it could represent a control group for a further experiment.***

**Response to the comment:**

The purpose of this study was to demonstrate the usefulness of the developed medical equipment for making distance between the target and surrounding organs and our spacer could be removed without surgery and not to demonstrate statistical significance.

Thank you for your comments and suggestions. We hope the revised manuscript is now suitable for publication in your journal.
